# Outcomes of Emergency Abdominal Wall Hernia Repair: Experience Over a Decade

**DOI:** 10.7759/cureus.26324

**Published:** 2022-06-25

**Authors:** GJ Pavithira, Souradeep Dutta, Sudharsanan Sundaramurthi, Vishnu Prasad Nelamangala Ramakrishnaiah

**Affiliations:** 1 Surgery, Jawaharlal Institute of Postgraduate Medical Education and Research, Puducherry, IND

**Keywords:** hernia repair, emergency hernia repair, femoral hernia, inguinal hernia, abdomen ventral hernia

## Abstract

Background

Abdominal wall hernias are a common surgical entity encountered by the general surgeon. Approximately 10% of abdominal wall hernia patients require emergency surgery. However, these surgeries are associated with a high rate of postoperative morbidity and mortality. This study aimed to analyze the morbidity and mortality in patients undergoing emergency abdominal wall hernia repair and to determine the factors associated with surgical site infection (SSI) and recurrence in these patients attending a tertiary care hospital in south India.

Methodology

Our study was a single-centered, 10-year retrospective and a one-year prospective study conducted in a tertiary care center in India. All patients who underwent emergency abdominal wall hernia repair between April 2009 and May 2020 were included. Patients’ demographic details, comorbidities, intraoperative findings, 30-day surgical outcomes including SSI, and recurrence were studied.

Results

Out of 383 patients in our study, 63.9% had an inguinal hernia, and 54% of the patients underwent tissue repair. SSI was the most common morbidity (21.9%). Postoperative sepsis was the only independent factor associated with perioperative mortality according to the logistic regression analysis (odds ratio = 22.73, p = 0.022).

Conclusions

Tissue repair for emergency hernia surgery has better outcomes than mesh repair in clean-contaminated cases.

## Introduction

Abdominal wall hernias are a common surgical entity encountered by the general surgeon. Abdominal wall hernias are broadly classified as groin hernias (inguinal, femoral, and obturator hernias) and ventral hernias (epigastric, umbilical, paraumbilical, Spigelian, and incisional). Approximately 10% of the patients with abdominal wall hernia require surgery in an emergency setting [1]. However, these surgeries are associated with a significant postoperative complication rate and poor prognosis compared to their elective counterparts [2].

The rates of surgical site infection (SSI), readmissions, and re-exploration are higher in emergency repair due to more frequent acute presentation with advanced comorbidities, including morbid obesity and poorly controlled diabetes. In addition to the comorbidities, patients’ general condition at presentation can pose further challenges. These challenges may include bowel obstruction causing acute inflammatory response leading to metabolic derangements, bowel ischemia or necrosis, and peritonitis following contamination [3]. All these can result in bacterial translocation, which decreases the threshold for infection. The use of mesh in an emergency surgery is still controversial despite the proven advantage in elective cases for its low hernia recurrence rates [4]. In the suboptimal setting, performing advanced techniques such as laparoscopy and component separation could be either less effective, or its role has not been well established in acute settings [2].

This study aimed to analyze the morbidity and mortality in patients undergoing emergency abdominal wall hernia repair retrospectively for 10 years and prospectively for one year. The outcome in patients undergoing emergency repair for abdominal wall hernia, the factors associated with complications such as SSI, and recurrence in patients undergoing emergency abdominal wall hernia repair were analyzed.

## Materials and methods

Our study was a single-center retrospective and prospective observational analytical study performed in the Department of Surgery, Jawaharlal Institute of Postgraduate Medical Education and Research, Puducherry, which is a tertiary care teaching hospital in south India. Data of all patients >18 years of age who underwent emergency abdominal wall hernia repair between April 2009 and April 2019 were collected retrospectively and from May 2019 to May 2020 were collected prospectively. The Institute Ethics Committee of the institute approved our study (JIP/IEC/2019/315). Written and informed consent was taken from all participants who were recruited prospectively.

The sample size was calculated to be 383 using Open EPI Version 3 with a prevalence of SSI among emergency hernia patients undergoing open hernia repair to be 10%, with a relative precision of 3% and confidence interval of 95% [5].

All participants in the retrospective group were contacted via telephonic calls/e-post to participate in further follow-up regarding outcomes and to obtain information regarding recurrence using the Ventral Hernia Recurrence Inventory [6].

The patients’ demographic characteristics, clinical findings, intraoperative, and 30-day postoperative outcomes, including the occurrence of SSI, pulmonary complications, ileus, sepsis, re-operation, readmission, and death, were studied. Our study utilized the United States Centers for Disease Control and Prevention (CDC) definition for the diagnosis of SSI and grading.

Statistical analysis was done using SPSS 21.0 software version for Windows (IBM Corp., Armonk, NY, USA). All the continuous variables not following normal distribution such as age, weight, surgery duration, hospital stay, and intensive care unit (ICU) stay were represented as the median and interquartile range (IQR) and were analyzed using the Mann-Whitney U test.

Categorical variables such as gender, comorbidities, American Society of Anesthesiologists (ASA) score, type of surgery, grades of SSI, presence of postoperative pneumonia, Clavien-Dindo classification, perioperative mortality, need for reoperation, and readmission were expressed as proportions and analyzed using the chi-square test and Fisher’s exact test after testing for normality using the Shapiro-Wilk test.

A logistic regression test was performed to determine the factors leading to SSIs and increased risk for mortality. Recurrence of hernia was analyzed using the Kaplan-Meier plot, and a log-rank test was used to study the statistical difference in hernia recurrence between the tissue repair (T) and mesh repair (M) groups.

## Results

A total of 383 patients were included in our study, of which 356 patients, who underwent emergency abdominal wall hernia repair between 2009 and 2019, were retrospectively studied, and 33 patients, who underwent emergency abdominal wall hernia repair from 2019 to 2020, were prospectively studied.

**Figure 1:**
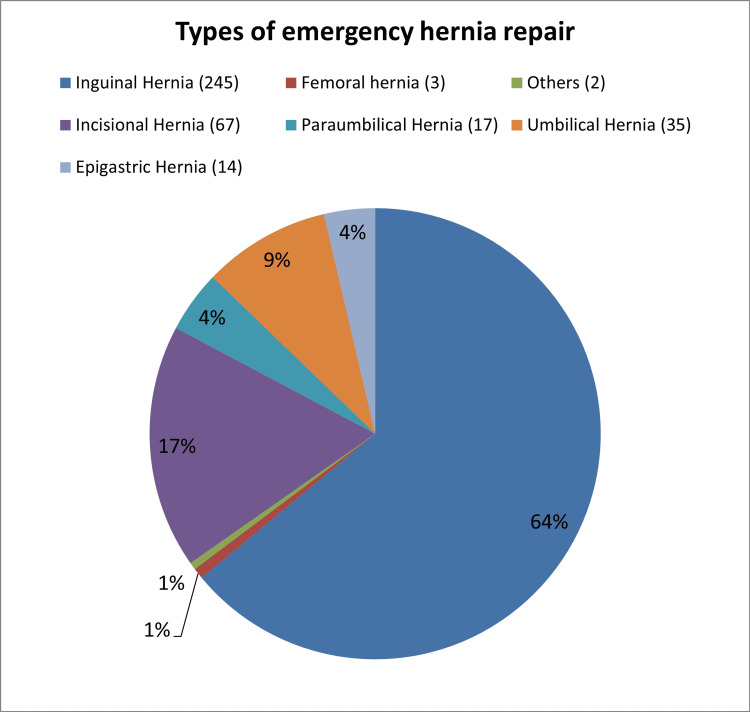
Types of emergency hernia repair.

The most common hernia in our study was inguinal hernia seen in 245 (63.9%) patients, followed by incisional hernia in 67 (17.5%) patients. A total of 66 (17.2%) patients had non-incisional ventral hernia which included small proportions of umbilical 35 (9.1%), para-umbilical 17 (4.4%), and epigastric hernia 14 (3.7%) (Figure 1).

Demographic data of the study population revealed that the median age was 55 (IQR = 45-65) years for both genders. The majority of the study population were males (males: female = 2.5:1). The median weight of the study population was 60 (IQR = 50-70) kg in both males and females. The most common comorbidity seen in our patients was hypertension (22.5%), followed by diabetes (11.5%). About 21.4% of the patients were immunocompromised with diabetes mellitus, bronchial asthma, and history of cancer or steroid use. A history of abdominal surgery was present in 22.7% of patients. Patients with higher incidence of comorbidities such as diabetes mellitus, coronary artery disease, chronic liver disease, and immunocompromised state underwent tissue repair more commonly than mesh repair. Tissue repair was done using interrupted 2-0 polyglactin or polypropylene sutures. Mesh repair was done with polypropylene mesh fixed using interrupted polypropylene stitches. The two groups did not differ in the history of abdominal surgeries or ventral hernia repairs (Table 1).

**Table 1:** Comparison of comorbidities in the tissue repair group and mesh group. *Chi-square test; ^#^Fischer test; ^ BPH proportion in male patients; ^†^Total number of patients mentioned in each group exceeds the actual number as more than one comorbidity was present in the same patient. CAD/CCF: coronary artery disease/congestive cardiac failure; COPD: chronic obstructive pulmonary disease; BPH: benign prostatic hyperplasia; TB: tuberculosis

Comorbidities	Total (n = 383^*^)	Tissue repair group (n = 207†)	Mesh group (n = 176†)	P-value*
Hypertension	86 (22.5)	52 (25.1)	34 (19.3)	0.175
CAD/CCF	23 (6)	18 (8.7)	5 (2.8)	0.016
Chronic liver disease	11 (2.9)	9 (4.3)	2 (1.1)	0.061
Chronic kidney disease	10 (2.6)	6 (2.9)	4 (2.3)	0.757^#^
COPD	12 (3.1)	6 (2.9)	6 (3.4)	0.775
Smoking	59 (15.4)	39 (18.8)	20 (11.4)	0.043
BPH^^^	56 (20.3)	30 (20.8)	2 (20.6)	0.946
History of pulmonary TB	11 (2.9)	7 (3.4)	4 (2.3)	0.476
Diabetes mellitus	44 (11.5)	30 (14.5)	14 (8)	0.046
Asthma	9 (2.3)	6 (2.9)	3 (1.7)	0.516^#^
Steroid use	20 (5.2)	13 (6.3)	7 (4)	0.313
Cancer	9 (2.3)	2 (1)	7 (4)	0.086^#^
Immunocompromised state±	82 (21.4)	52 (19.8)	24 (11.9)	0.037
Past abdominal surgeries	87 (22.7)	42 (20.3)	45 (25.6)	0.219
Past ventral hernia repair	25 (6.5)	15 (7.2)	10 (5.7)	0.537
Past mesh repair	10 (2.6)	4 (1.9)	6 (3.4)	0.663

More patients with the clinical findings of peritonitis and ascites at presentation underwent tissue repair than mesh repair (p = <0.001 and 0.005, respectively). Majority of the study population belonged to ASA grade I (41.8%) while only a minor proportion belonged to ASA physical status IV (4.7%). Higher number of patients with ASA grade I underwent mesh repair (T = 74, M = 86; p = 0.009) while more patients with ASA grade IV underwent tissue repair (T = 15, M = 3; p = 0.011). Most patients (n = 144) presented with intestinal obstruction, followed by irreducibility of hernia (n = 139). About 250 (65.3%) patients had bowel as the hernial sac content, of whom 96 (25.1%) patients had strangulated bowel intraoperatively (Table 2).

**Table 2:** Comparison of perioperative variables in the mesh and tissue repair groups. *Chi-square test; ^†^ Mann-Whitney U test; ^#^ Fisher’s exact test. ASA: American Society of Anesthesiologists physical status classification system; IQR: interquartile range

Preoperative variables	Tissue repair group (n = 207)	Mesh group (n = 176)	Total (n = 383)	P-value^*^
Presence of signs of peritonitis	27 (13.04)	4 (2.3)	31 (8.1)	<0.001
Presence of ascites	21 (10.1)	5 (2.8)	26 (6.8)	0.005
ASA grade				
I	74 (35.7)	86 (48.9)	160 (41.8)	0.009
II	70 (33.8)	59 (33.5)	129 (33.7)	0.952
III	48 (23.2)	28 (15.9)	76 (19.7)	0.075
IV	15 (7.2)	3 (1.7)	18 (4.7)	0.011
Surgery duration in minutes (IQR)	120 (90–150)	105 (60–120)	^-^	0.029^†^
Size of the defect in cm (IQR)	2.4 (2–3)	2.2 (1.9–4)	2.4 (2–3)	0.734^†^
Hernia complication				
Irreducible	56 (27.1)	83 (47.2)	139 (36.3)	<0.001
Obstructed	72 (34.8)	72 (40.9)	144 (37.6)	0.217
Strangulated	76 (36.7)	20 (11.4)	96 (25.1)	<0.001
Eviscerated	3 (1.4)	1 (0.6)	4 (1)	0.628^#^
Sac content				
Small bowel	111 (53.6)	72 (40.9)	183 (47.8)	0.013
Large bowel	17 (8.2)	26 (14.8)	43 (11.2)	0.043
Small and large bowel	15 (7.2)	4 (2.3)	19 (5)	0.025
Omentum	36 (17.4)	41 (23.3)	77 (20.1)	0.151
Preperitoneal fat	8 (3.9)	1 (0.6)	9 (2.3)	0.042^#^
Appendix	2 (1)	3 (1.7)	5 (1.3)	0.665^#^
Bladder	3 (1.4)	0 (0)	3 (0.8)	0.253^#^
None	15 (7.2)	29 (16.5)	44 (11.5)	0.005
Reactive fluid	48 (23.2)	28 (15.9)	76 (19.8)	0.075

A total of 69 (18%) patients underwent resection for either strangulation or the presence of pre-gangrenous changes in the bowel. Patients with strangulation of bowel mostly underwent tissue repair (T = 76, M = 20; p < 0.001), unlike those who had only an irreducible hernia who predominantly underwent mesh repair (T = 56, M = 83; p < 0.001) (Table 3).

**Table 3:** Comparison of hernia variables and bowel characteristics in mesh and tissue repair groups * Chi-square test.

Nature of bowel	Tissue repair group (n = 145)	Mesh group (n = 105)	Total (n = 250)	P-value^*^
Normal	54 (37.2)	90 (85.7)	144 (57.6)	<0.001
Congested	18 (12.4)	6 (5.7)	24 (9.6)	0.033
Pre-gangrenous	6 (4.1)	0 (0)	6 (2.4)	0.033
Patchy gangrenous	6 (4.1)	0 (0)	6 (2.4)	0.033
Gangrenous	61 (42)	9 (8.6)	70 (28)	<0.001
Bowel resected	64 (44.1)	5 (4.7)	69 (27.6)	-
None	81 (55.8)	100 (95.2)	181 (72.4)	<0.001
Small bowel	57 (39.3)	4 (3.8)	61 (2.4)	<0.001
Large bowel	3 (2.1)	0 (0)	3 (1.2)	0.253
Both small and large bowel	3 (2.1)	1 (1)	4 (1.6)	0.628
Appendix	1 (0.7)	0 (0)	1 (0.4)	0.391

Intraoperatively, drains were placed in 29.5% of the patients. Most patients with a drain belonged to the tissue repair group when compared to the mesh repair group (p = 0.03). The median duration of surgery was significantly longer in the tissue repair group [120 (IQR = 90-150) minutes] when compared to the mesh group [105 (IQR = 60-120) minutes] (p = 0.029).

In the postoperative period, the most common complication was SSI (21.9%), followed by pulmonary complications (11.7%) and urinary tract infections (3.4%). Between the two groups, patients in the mesh repair group had a significantly lower incidence of SSI (T = 53, M = 30; p = 0.033). Patients in the tissue repair group developed postoperative sepsis more commonly than those in the mesh repair group (T = 4.3%, M = 0.6%; p = 0.024). Patients in the tissue repair group required prolonged ICU stay and hospital stay when compared to those in the mesh repair group, the difference of which was statistically significant. A total of 10 patients, five in each group, underwent re-exploration in the postoperative period (Table 4).

**Table 4:** Comparison of postoperative complications in the mesh and tissue repair groups. ^#^ Fisher’s exact test; ^†^ Mann-Whitney U test; * Chi-square test. ICU: intensive care unit; IQR: interquartile range; LRTI: lower respiratory tract infection; VAP: ventilator-associated pneumonia

Postoperative complications	Tissue repair group (n = 207)	Mesh group (n = 176)	Total (n = 383)	P-value^*^
Surgical site infection	53 (26.1)	30 (17)	83 (21.9)	0.033
Pulmonary complications	30 (14.5)	15 (8.5)	45 (11.7)	0.117
LRTI	7	5	12	0.762
Atelectasis	16	7	23	0.123
Pleural effusion	5	2	7	0.352
VAP	2	1	3	1.000
Ileus	12 (5.8)	5 (2.8)	17 (4.4)	0.162
Postoperative fistula	1 (0.5)	1 (0.6)	2 (0.5)	1.000
Urinary tract infection	9 (4.3)	4 (2.3)	13 (3.4)	0.264
Cardiac complications	5 (2.4)	6 (3.4)	11 (2.9)	0.562
Renal failure requiring dialysis	3 (1.4)	2 (1.1)	5 (1.3)	1.000
Liver failure	3 (1.4)	1 (0.6)	4 (1)	0.628
Seroma	2 (1)	2 (1.1)	4 (1)	1.000
Presence of sepsis	9 (4.3)	1 (0.6)	10 (2.6)	0.024^#^
Days of ICU stay median (IQR)	2 (2–3)	1.5 (1–2)	2 (1–3)	0.009^†^
Days of hospital stay median (IQR)	8 (5–11)	6 (4–9)	7 (5–10)	0.006^†^
30-day re-admission (%)	1 (0.5)	1 (0.6)	2 (0.5)	1.000^#^
Reoperation (%)	5 (2.4)	5 (2.8)	10 (2.6)	1.000^# ^
Mortality	4 (1.9)	2 (1.1)	6 (1.6)	0.528

More patients with clean surgical wounds underwent mesh repair whereas patients with contaminated and dirty surgical wounds predominantly underwent tissue repair (Table 5).

**Table 5:** Association between CDC wound classification and surgical site infection between the tissue and mesh repair groups * Chi-square test; ^#^ Fisher’s exact test. CDC: Centers for Disease Control and Prevention; SSI: surgical site infection

CDC classification	SSI	Tissue repair (n = 207)	Mesh repair (n = 176)	P-value^*^
Class 1: Clean	Present	17 (8.2)	20 (11.4)	0.537
	Absent	72 (34.8)	106 (60.2)	
	Total	89 (43)	126 (71.6)	<0.001
Class II: Clean- contaminated	Present	17 (8.2)	8 (4.5)	0.570
	Absent	60 (28.9)	37 (21)	
	Total	77 (37.2)	45 (25.6)	0.015
Class III: Contaminated	Present	9 (4.3)	0 (0)	0.268^#^
	Absent	14 (6.8)	4 (2.3)	
	Total	23 (11.1)	4 (2.3)	0.001
Class IV: Dirty infected	Present	11 (5.3)	0 (0)	1.000^#^
	Absent	7 (3.4)	1 (0.6)	
	Total	18 (8.7)	1 (0.6)	<0.001

Morbidity and mortality were studied using the Clavien-Dindo classification. A significantly higher number of patients in the mesh group belonged to Clavien-Dindo class I (T = 6, M = 14; p = 0.027) whereas most patients who underwent tissue repair were in classes II- V (T = 201, M = 162).

Mortality was present in six (1.6%) patients in our study population. There was no significant difference in mortality rate between the tissue and mesh repair groups (1.9% vs. 1.1%; p = 0.528). One patient in the mesh group had acute myeloid leukemia and expired in the postoperative period because of medical complications rather than surgical complications. All four mortalities in the tissue repair group and one in the mesh repair group developed postoperative sepsis.

The factors associated with postoperative SSI, in patients who underwent emergency abdominal wall hernia, by univariate analysis, were diabetes mellitus, past abdominal surgery, presence of peritonitis and ascites, bowel resection, surgery duration exceeding two hours, and CDC wound class I and IV. According to multivariate analysis, patients who underwent surgery for more than two hours had 2.6 odds of developing SSI (p = 0.003, 95% CI = 1.371-4.956). Similarly, CDC class IV wound patients had 3.83 odds of developing SSI (p = 0.032, 95% CI = 2.950-21.615). Perioperative mortality was affected by the presence of peritonitis, ascites, bowel resection, drain placement, re-exploration, postoperative pulmonary complications, and sepsis in univariate analyses. However, following a multivariate analysis, sepsis was found to be the sole factor affecting mortality in these patients (p = 0.022, 95% CI = 1.574-328.293) (Table 6).

**Table 6:** Association factors for perioperative SSI and perioperative mortality in abdominal hernia repair patients. CDC: Centers for Disease Control and Prevention; SSI: surgical site infection

	Perioperative SSI			Perioperative mortality		
Parameters	Present (n = 83)	Absent (n = 300)	P-value	Present (n = 6)	Absent (n = 377)	P-value
Diabetes mellitus (n = 44)	16 (19.2)	28 (9.3)	0.014	-	-	-
Past abdominal surgery (n = 87)	27 (32.5)	60 (20)	0.020	-	-	-
Surgery duration >2 hours (n = 104)	40 (48.2)	64 (21.3)	0.000	-	-	-
CDC classification				-	-	-
Clean class I (n = 215)	37 (44.6)	178(59.3)	0.017	-	-	-
Dirty class IV (n = 19)	12 (14.4)	7 (2.3)	<0.001	-	-	-
Presence of peritonitis (n = 31)	13 (15.6)	18 (6)	0.005	3 (50)	28 (7.4)	0.008
Presence of ascites (n = 26)	11 (13.2)	15 (5)	0.009	2 (33.3)	24 (6.3)	0.056
Bowel resection (n = 69)	27 (32.5)	42 (14)	0.000	4 (66.7)	65 (17.2)	0.022
Drain placement (n = 113)	-	-	-	5 (83.3)	108 (28.6)	0.011
Re-exploration(n = 10)	-	-	-	2 (33.3)	8 (2.1)	0.009
Postoperative pulmonary complication (n = 45)	-	-	-	4 (66.7)	41 (10.9)	0.003
Postoperative Sepsis (n = 10)	-	-	-	4 (66.7)	6 (1.6)	<0.001

**Figure 2:**
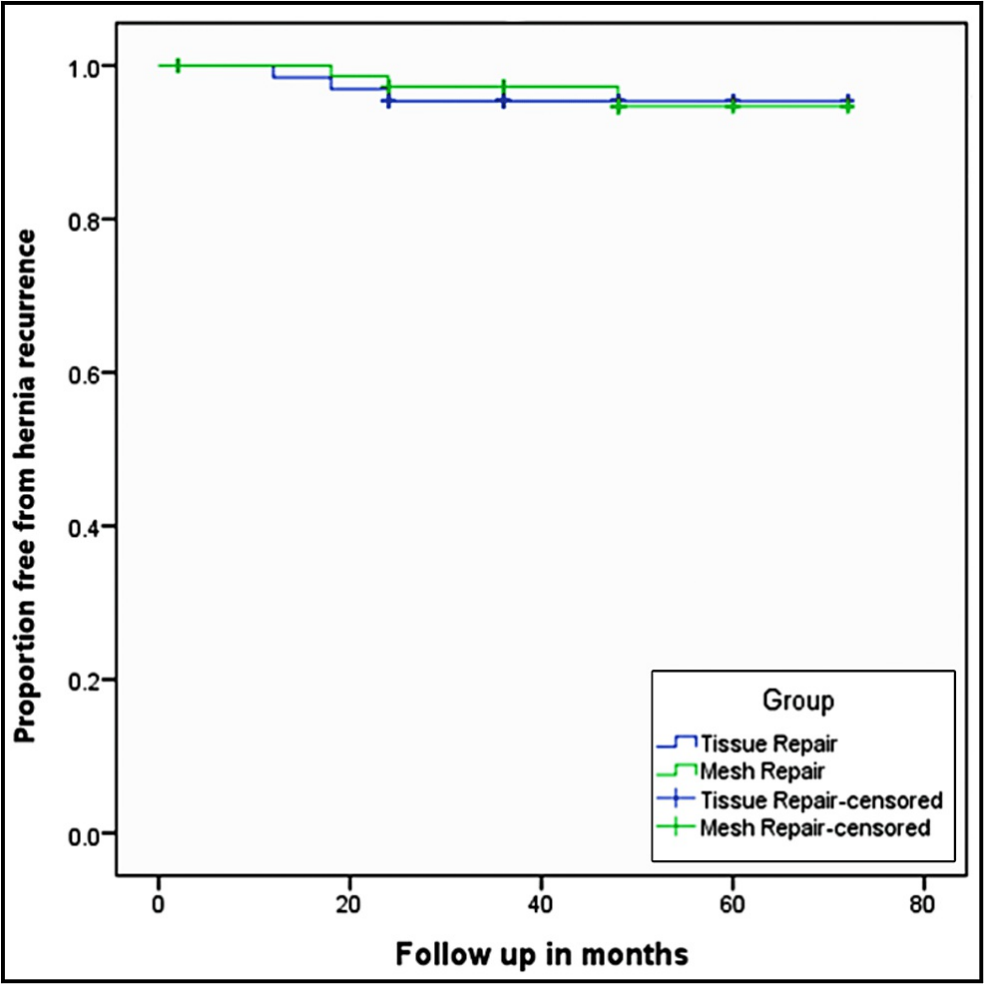
The Kaplan-Meier plot of recurrence after abdominal wall hernia repair.

The overall recurrence rate of hernia in our study was 3.6%. Recurrence rate was more in the mesh group (4.8%) when compared to the tissue group (1.6%) with a p-value of 0.056. The Kaplan-Meier plot estimate for recurrence in emergency abdominal ventral hernia repair patients is shown in Figure 2.

We could follow 165 patients to study the recurrence pattern. The flat curves indicate a low rate of recurrence of hernias. Though recurrence was more in the mesh repair group, it was not significant (p = 0.786).

## Discussion

In our study, inguinal hernia was the most commonly encountered entity requiring emergency hernia repair. About 245 (63.9%) patients had an inguinal hernia of which 225 (91.8%) were seen in males, which was similar to the survey conducted by Venara et al., where 65.1% had inguinal hernia [7].

In women, the most common type of hernia identified in our study was incisional hernia (55.6%). The higher incidence of incisional hernia in women could be due to obstetric surgical history with obesity and lax abdomen post-pregnancy. Improper emergency settings for cesarean section surgeries along with a higher incidence of anemia and malnutrition in developing countries can amount to an increased risk of developing incisional hernia in women [8].

In our study, only three patients had a femoral hernia (0.8%), and all three presented with intestinal obstruction. In a nationwide register-based study, where 46,717 groin hernia repairs were studied, the incidence of femoral hernia repair was 3%. In one of the Indian studies by Sulaiman et al., four out of 157 patients had a femoral hernia (2.5%) [9]. The risk factors for the development of femoral hernia include female gender, multiple pregnancies, increased intra-abdominal pressure like chronic constipation, and increasing age.

Around 57% of our study population had one or more comorbidities, which increased to 77% in patients over 65 years of age. Patients with comorbidities underwent tissue repair more commonly in our study (T = 68.4%, M = 31.6%). Haskins et al., in a retrospective study, investigated 2,449 patients with a ventral hernia and found that patients with diabetes mellitus underwent tissue repair more commonly (p = 0.02) [10].

Patients with clean surgical wounds underwent mesh repair more frequently (M = 126, T = 89), whereas patients with clean-contaminated (T = 77, M = 45), contaminated (T = 23, M = 3), and dirty wounds (T = 18, M = 1) underwent tissue repair predominantly. In the literature, evidence shows that patients undergoing mesh repair with a clean-contaminated or contaminated surgical field have an increased incidence of SSI compared to patients with a clean surgical area [11]. The World Society of Emergency Surgery (WSES) 2017 update advised mesh use in clean-contaminated cases because bowel strangulation or the need for bowel resection in the absence of contamination did not increase the chance of SSI [4]. In our study, we placed mesh in 45 patients out of the 122 patients who had a clean-contaminated surgical field. Mesh placement was mainly influenced by the operating surgeons’ decision intraoperatively to avoid mesh infection. Bessa et al. studied 234 patients who underwent emergency ventral hernia repair and noted that the only limitation for mesh use was gross contamination of the surgical field [12].

In our study, the duration of surgery was significantly higher in the tissue repair group. Similar findings were observed in the meta-analysis by Grant et al., where the time taken to perform mesh repair was shorter than other non-mesh repairs [13]. We attributed the longer surgery duration in the tissue repair group to patients with obstruction or strangulation. These patients warranted hyper-oxygenation and warm mops for either congested, pre, or patchy gangrenous bowel, which could have potentially increased the intraoperative time. Moreover, patients who had gangrenous bowel had to undergo resection, which prolonged the surgery duration overall.

Derici et al., in their study on incarcerated abdominal wall hernia patients, noticed that strangulation was present in 42.9% of their study population [14]. Similar research done by Emile et al. had 57.4% of patients with bowel strangulation [15]. In our study, 96 (25.1%) patients presented to the emergency with strangulation, and 79.2% of them underwent tissue repair. Compared to these studies, where only incarcerated ventral hernias were included, our study also evaluated patients with uncomplicated hernias done in the emergency.

Bessa et al., in a 10-year retrospective study on emergency management of complicated groin hernias with mesh, concluded that it was safe to use prosthetic mesh in patients presenting with strangulation and incarceration [12]. In our research, surgeons preferred tissue repair to mesh placement in these patients, particularly in the elderly and those with comorbidities, because the chance of postoperative SSI was higher in our emergency setting (34.1%).

The small bowel was the most common content of the hernial sac in our study population (52.7%). Similarly, Tastaldi et al., in a 10-year retrospective study of emergency hernias, noted that a majority (55.3%) of their population had small bowel as hernial sac content [16].

In our study, 69 (18%) patients underwent bowel resection for complications such as gangrene, of whom only five patients underwent mesh repair. Derici et al., in their study, observed that 19.2% of their patients required bowel resection [14]. A systemic review by Hentati et al. did not recommend using mesh after bowel resection in incarcerated hernia patients [17]. The mesh placement was limited intraoperatively by factors such as contamination of the surgical field, resection of the bowel, and, more importantly, the heterogeneous surgical approach by the operating surgeons in our study.

In our study, 21.9% of the patients had SSIs. The incidence of SSI in tissue and mesh repair patients was 26.1% and 17%, respectively. In a study by Nieuwenhuizen et al., 203 patients underwent acute hernia repair, and the overall SSI rate was 12.3% [18]. In one of the Indian studies by Keswani et al., where 198 patients with abdominal wall hernia were analyzed, SSI was observed in 7% of elective surgeries and in almost 50% of emergency surgeries [19]. Emile et al. observed that the SSI rate was higher (7.5%) in the mesh group than in the tissue repair group (5.3%) [15]. Pandey et al. in their study noticed that the SSI rate was 26% in contaminated cases [20]. In our study, the prolonged surgery duration and contaminated surgical fields in tissue repair groups could explain the higher SSI rate.

WSES consensus suggests using mesh in clean-contaminated cases as it does not increase the postoperative wound morbidity [4]. In our study, eight out of 45 patients (17.8%) with clean-contaminated surgical fields who underwent mesh repair developed SSI whereas among the 77 patients who underwent tissue repair for clean-contaminated cases the SSI rate was 22.07%. Although the WSES consensus advocates mesh repair in clean-contaminated cases, more patients with clean-contaminated wounds underwent tissue repair than mesh repair (77 vs. 45) in our study. This could be because of the surgeon’s decision based on intraoperative findings and the clinical condition of the patient.

The incidence of pulmonary complications was 11.7%, which was the second most common postoperative complication in the present series. The incidence was comparable to that noted by Martinez Serrano et al. (9.6%) in their analyses of 402 patients who underwent emergency surgery for abdominal wall hernias [21]. The pulmonary complication rate in the study by Venara et al. was less (2.4%) when compared to our study as they had included only patients with pneumonia [7]. In our study, all patients with pneumonia and those with atelectasis, pleural effusion, and lower respiratory tract infections were also included.

The incidence of sepsis in the postoperative period was 2.6% in our study and it was 2.3% in the study by Tastaldi et al [16]. In our study, out of the 10 patients with sepsis, five patients had respiratory complications, and four patients had an anastomotic leak. One patient had both pulmonary complications and an anastomotic leak that required re-exploration. Out of the six patients with sepsis in the study by Tastaldi et al., four had pneumonia; one patient each had a bloodstream infection and urinary tract infection [16]. The respiratory complication was a common factor leading to sepsis in both the study populations.

Postoperative complications were more common among patients who underwent tissue repair than those who underwent mesh repair. In our study, 63.9%, 70.6%, and 90% of patients with SSI, postoperative ileus, and sepsis, respectively, underwent tissue repair. The hospital stay duration was significantly longer for the tissue repair group patients (eight vs. six days; p = 0.006). An increase in hospital stay duration in the tissue group may be due to increased postoperative complications compared to the mesh group.

Mortality was present in six (1.6%) patients in our study population. There was no significant difference in mortality rate between the tissue and mesh repair groups (1.9% vs. 1.1%; p = 0.53). One patient in the mesh group had acute myeloid leukemia and expired in the postoperative period because of medical complications rather than surgical complications. All four mortalities in the tissue repair group and one in the mesh repair group developed postoperative sepsis. Gul et al., in their study on 131 patients with incarcerated abdominal wall hernia, observed an overall mortality rate of 2.1%, but they noted a significant difference between mesh repair and tissue repair group (0% vs. 5.9%; p < 0.028) [22].

Emile et al. observed that patients with diabetes mellitus, previous hernia surgery, intestinal resection, and clean-contaminated wounds were associated with SSI [15]. Campbell et al. showed that prolonged surgery had increased SSI incidence postoperatively considering the complexity of the cases [23]. In our study, we found that increased surgery duration of more than two hours and a dirty surgical field (CDC class IV) were the two independent factors associated with SSI.

Our study showed that the presence of peritonitis, bowel resection, drain placement, pulmonary complication, and postoperative sepsis were the factors associated with perioperative mortality. Sepsis was found to be the only independent factor affecting mortality by univariate and multivariate analyses. Derici et al. investigated the factors associated with morbidity and mortality in incarcerated abdominal wall hernia patients. They found that among the various factors significant by univariate analyses, necrotic bowel was the single most important factor associated with morbidity and mortality by multivariate analyses. Both in our study and the study by Derici. et al. [14], gangrenous bowel requiring resection was an essential factor affecting the mortality in patients who had sepsis.

The overall hernia recurrence rate was 3.6% in our study. The hernia recurrence rate in patients who underwent mesh repair was 4.8%, whereas only 1.6% of the patients who underwent tissue repair had recurrence with no significant difference. Previous studies in the literature showed that the recurrence rate was more common in the tissue group than in the mesh group [17]. In our study, the unavailability of data from the past years and inadequate follow-up of patients could render an unreliable result on the recurrence rate.

Limitations

This study has its inherent limitations due to its retrospective nature, such as missing data and underestimated complications. Further, the treatment decisions were made by different surgeons, leading to a heterogeneous surgical treatment. As there were no existing guidelines for mesh use, our study intent was to provide a current institutional practice. A cautious interpretation of the various risk factors identified by univariate analysis for SSI and mortality is needed as there were many other confounding factors. There were insufficient data available on the long-term follow-up, so we could not conclude the association between mesh use and higher recurrence rates. Nonetheless, in the controversial field of hernia surgery, our institutional practices, along with a well-maintained and analyzed audit, will contribute to the already existing evidence in the literature, which is still considered difficult to obtain and scarce.

## Conclusions

In our emergency hernia surgery practice, SSI was high and was the most common morbidity. Our study found that prolonged surgery and dirty surgical wounds were the main factors associated with SSI. The sole factor independently associated with mortality in our study was sepsis. Hence, mesh use, in a clean-contaminated wound should be decided by the operating surgeon on a case-by-case basis. Mesh use can be avoided when the risk of SSI is high as in elderly patients with uncontrolled comorbid conditions. Although the majority underwent tissue repair, the recurrence rate in them was low in our study. Therefore, we conclude that tissue repair for emergency hernia surgery has better outcomes than mesh repair.
